# Accessibility of osteochondral lesion at the capitellum during elbow arthroscopy: an anatomical study

**DOI:** 10.1007/s00402-023-05172-7

**Published:** 2024-01-03

**Authors:** S. Wegmann, M. Hackl, F. Krane, K. Wegmann, L.-P. Mueller, T. Leschinger

**Affiliations:** 1 Faculty of Medicine and University Hospital, Center for Orthopedic and Trauma Surgery, University Hospital of Cologne, Kerpener Str. 62, 50937 Cologne, Germany; 2 OCM (Orthopädische Chirurgie München) Clinic, Steinerstr. 6, 81369 Munich, Germany

**Keywords:** Osteochondrosis dissecans, Elbow, Arthroscopy, Nanodrilling

## Abstract

**Introduction:**

Osteochondrosis dissecans (OCD) at the capitellum is a common pathology in young patients. Although arthroscopic interventions are commonly used, there is a lack of information about the accessibility of the defects during elbow arthroscopy by using standard portals.

**Materials and methods:**

An elbow arthroscopy using the standard portals was performed in seven fresh frozen specimens. At the capitellum, the most posterior and anterior cartilage surface reachable was marked with K-wires. Using a newly described measuring method, we constructed a circular sector around the rotational center of the capitellum. The intersection of K-wire “A” and “B” with the circular sector was marked, and the angles between the K-wires and the Rogers line, alpha angle for K-Wire “A” and beta angle for K-wire “B”, and the corridor not accessible during arthroscopy was digitally measured.

**Results:**

On average, we found an alpha angle of 53° and a beta angle of 104°. Leaving a sector of 51° which was not accessible via the standard portals during elbow arthroscopy.

**Conclusion:**

Non-accessible capitellar lesions during elbow arthroscopy should be considered preoperatively, and the informed consent discussion should always include the possibility of open procedures or the use of flexible instruments.

**Level of Evidence:**

4.

## Introduction

Osteochondrosis dissecans (OCD) is a localized disease of joints that usually develops during growth and is part of aseptic bone necrosis. In the process vitality of the subchondral bone is locally impaired, causing a delamination and sequestration of subchondral bone with or without influence on the overlying cartilage [1]. Reports of a prevalence of 15–30 per 100,000 have been made, especially in athletes participating in sports exerting repetitive strain on the radiocapitellar joint [2, 3]. This leads to the elbow joint being the second most affected joint after the knee and OCD of the elbow representing 6% of all osteochondral lesions [4]. Juvenile OCD mainly affects young patients between 10 and 20 years and is a leading cause of permanent elbow disability in adolescents and young adults [5]. Adult OCD affects skeletally mature patients and generally has a poorer prognosis. The aetiology is still poorly understood and there is an ongoing scientific discourse, but trauma and perfusion disorders play a significant role [6]. Histological analysis showed that the pathologic progressions depend on the underlying cause of OCD [7]. In case of a primarily vascular cause, Campbell, Ranawat et al. suggested regional vascular insufficiency as a common aetiology [8]. If trauma was the underlying cause, subchondral fractures were found and led to bone necrosis [7]. Male gender and extreme obesity seem to be risk factors [3, 9].

Typically, patients present with insidious pain in the lateral elbow which, is related to or aggravated by exertion. Additionally, an extension deficit, a blocked joint and crepitations can be seen. Because of insufficient specificity, further radiological examination with MRI scan is obligatory. In case of surgery, the most used procedures are arthroscopic interventions like arthroscopic debridement and microfracturing/nanodrilling which was first described in 1957 by Smilie et al. and later evaluated by Steadman and his group or arthroscopic fragment fixation [10, 11].

The first classification of OCD was done in 1959 by Berndt and Harty and established a four-stage classifications system based on the severity of lesions in plain radiographs on osteochondral lesions of the talus [12]. For the elbow, the classification of Minami et al. is generally accepted [13]. Yet, an MRI-scan remains the gold standard to evaluate the size of the lesion, exact localisation, and stability. Commonly, the classification of Dipaola et al. is used as it differentiates four stages [14]. But also, the previously for the knee joint invented arthroscopic classification of the International Regeneration & Joint Preservation Society (ICRS) is used for the elbow [15].

Treatment options range from a conservative treatment, especially used in stable lesions grade I after Dipaola/ICRS, to operative treatment options. Here arthroscopic or open procedures are available. For instable lesions grade II after Dipaola/ICRS II-III with osteochondral fragments Nobuta et al. suggest refixation using Herbert screws, K-wires, or degradable pins [16]. Another treatment possibility lies in the use of osteochondral autologous transfer system (OATS). The technique can be used for instable lesions grade III/IV after Dipaola with destruction of more than 50% of the capitellum and a depth of up to 15 mm [17]. Other techniques of cartilage transfer like autologous chondrocyte transplantation (ACT) are also available options [18].

Due to its minimally invasive character and the improved understanding of portal placement and neurovascular structures leading to a reduced complications risk arthroscopy is recommended as a first-line operative treatment [19–21].

Even though anatomical studies have shown the feasibility of the elbow joint for arthroscopic techniques in the past, due to a small working space and adjacent neurovascular structures, visualization of the joint remains an ongoing issue and gives room for improvement [22, 23]. In a foregone study by Trofa et al. it was shown, that 66.5% of the capitellum can be visualised during a standard elbow arthroscopy, the accessibility regarding the treatment options like micro-fracturing was not evaluated [24]. In our clinical experience and also studies have shown, the incomplete visualization and especially the accessibility of the capitellum continue to be a major challenge leading to poor patient outcomes, even though flexion and extension can provide a remedy [25].

The aim of this anatomic study was to evaluate the capitellum for its accessibility during elbow arthroscopy by using standard portals. Further, we wanted to suggest a standardized method on the basis of plain radiographs to preoperatively evaluate if an OCD lesion is accessible during arthroscopy.

## Materials and methods

The study was approved by the local ethics committee (22-1451).

For the present anatomical study, seven elbows of fresh frozen specimens (four male, three female) were available. Specimens were excluded if there was evidence of previous injuries, relevant arthritic deformities, or previous operative treatments to the elbow joint.

The mean age of the donors was 74,8 with SD of 13,6 years (range: 58–90 years). The specimens were detached from the torso, and after thawing, fixed proximally by an arm holder creating a 90° flexion in the elbow joint with the hand facing the floor, simulating operative conditions as during a standard lateral technique for elbow arthroscopy.

Following, the proximal posterolateral portal (PLP), the direct lateral portal (DLP), the proximal anterolateral (PALP), and the anteromedial portal (AMP) were created as described by Graves et al. [26].

**Fig. 1:**
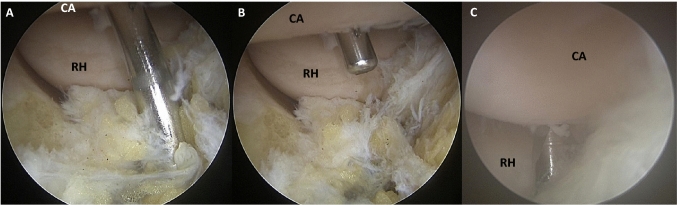
The **A** + **B** View from the PLP inserting the K-Wire using the DLP. The radial head (RH) and the capitellum (CA) can be clearly visualized. **C** View from AMP and marking the most anterior capitellar surface in maximum extension using the PALP

A diagnostic arthroscopy of the elbow joint was performed with visualisation of the capitellar surface. (Fig. 1). The elbow was brought to maximum flexion and the joint was visualised over the PLP. The most posterior cartilage-covered articular surface that is reachable by a K-wire using the DLP was visualised and marked with the K-wire (“A”) (Fig. 1 picture A & B). The same was done visualising the joint via the AMP and marking the most anterior capitellar surface in maximum extension using the PALP (Fig. 1 picture C). Thus, marking the boundaries of the articular surface not reachable with a K-wire (“B”) during arthroscopy.

Afterwards, a strict lateral radiograph was made, and care was taken that the humeral condyles were overlapping and the humeroulnar joint space was freely visible. The radiographs were digitally stored and evaluated using a digital image analysis software (ImageJ software, http://imagej.net). For the calibration of the imaging and the determination of the rotational center of the capitellum, the digital drawing of Rogers’ line (R) and the radiocapitellar line (RC) was carried out [27, 28]. The intersection of these two lines marks the rotational center of the capitellum and served as the starting point and center for drawing a circle. This circle was enlarged with a radius (r) to match the image size, creating a full circle, as is typical with a protractor, which measures 360°. The intersection between the circle and Rogers’ line was assumed to be 0° or 360°. The angle at which K-wire "A" (angle alpha) and "B" (angle beta) intersect with the circle were each measured and documented. Thus, creating a sector between the two K-wires or their angles on the full circle that cannot be reached by arthroscopy.

The same method can be used for the preoperative evaluation of an OCD lesion based on sagittal slices of MRI or CT. The calibration and application of the full circle are the same. Then, starting from the capitellar rotation center, the superior and inferior lesion boundaries are marked with a line. Similar to the intersections of the K-wires with the full circle, the intersections of these lines now serve to determine the extent of the lesion in degrees on the capitulum.

**Fig. 2:**
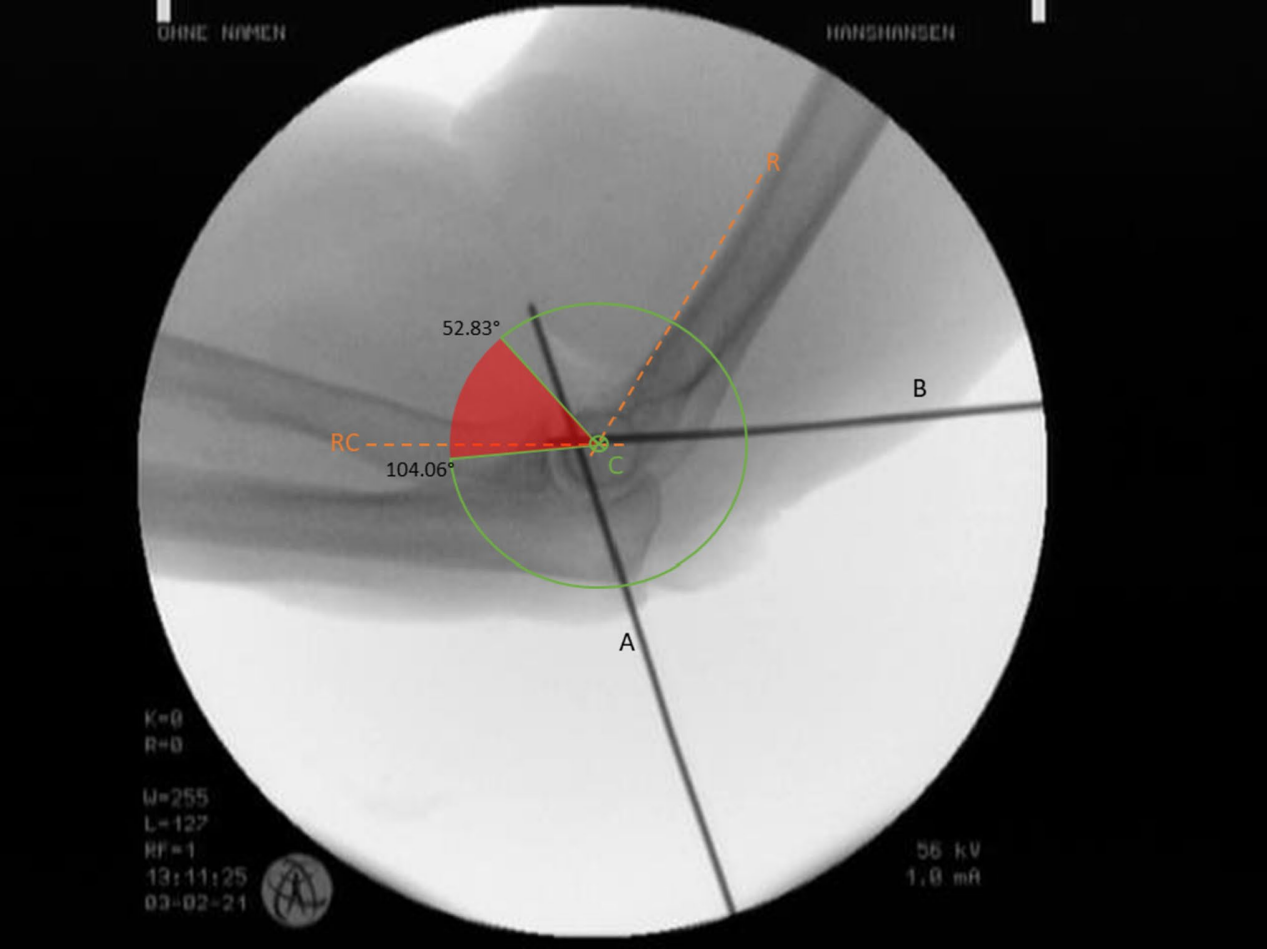
Lateral radiograph with the rogers line (R) and the radio capitellar line (RC) intersecting in the center of rotation (**C**). The intersection of the capitellar surface with both K-wires (**A** and **B**) is marked and the angle between R and the intersection is digitally measured, leaving the corridor which is not reachable (red surface) in between

An example is presented in Fig. 2.

## Results

On average, an alpha angle of 52.8° and a beta angle of 104.1° was measured, leaving a sector of 51.3° which is not accessible via the standard portals during elbow arthroscopy. In Table 1, the descriptive results of our study are presented.

**Table 1:** Descriptive results of the study

	Alpha angle	Beta angle
Median	52.8°	104.1°
Minimum	31.1°	68.5°
Maximum	76.3°	126.8°
Standard deviation	15.3°	20.4°

## Discussion

The results show that elbow arthroscopy using the standard portals has its limitations in reaching the anterior aspect of the capitellar surface using inflexible instruments leaving a sector of 51°, which was not accessible in our study.

The sector not accessible with K-wires for example in case of planned nanodrilling can possibly be evaluated preoperatively based on plain radiographs or with sagittal slices of MRI or CT scans by the method described in this study, thus aiding in the decision-making whether an arthroscopic treatment is a viable option or open operative treatments must be pursued or at least the patient educated on in the informed consent.

Due to its minimally invasive character and the improved understanding of portal placement and neurovascular structures leading to a reduced complications risk, arthroscopy is recommended as a first-line surgical approach if possible [19–21]. The current literature manifest for example, that arthroscopic debridement and nanodrilling or microfracturing for OCD treatment has good clinical outcomes. Bexkens and his study group investigated 71 patients for a mean follow-up of 3.5 years and were able to demonstrate good results regarding elbow pain, function, range of motion, with only 62% of the patients returning to their primary sport [29]. Lewine and her colleagues could confirm these results and could show that on average the elbow flexion improved by 12 degreed and extension by 21 degrees [30]. A meta-analysis including 11 studies and 327 patients by McLaughlin and his team showed, that debridement and nanodrilling had excellent results in improving pain, range of motion, patient outcome scores, and return to sport, yet regarding mid-term outcomes, debridement alone could produce comparable results without the use of nanodrilling in small defects [31]. Additionally, if necessary, refixation of a fragment could also be performed arthroscopically with a good clinical outcome [32]. Especially when an orthogonal approach is necessary for the treatment of osteochondritis dissecans, and the lesions must be reattached using K-wires or absorbable pins, or when a cartilage replacement procedure such as OATS is used, arthroscopy reaches its limits [33, 34]. Besides improved visualization, access for the instruments must also be ensured. Our results could show that arthroscopy of the elbow in the treatment of osteochondrosis dissecans has limitations in the accessibility of the entire articular surface. Even though Trofa and his colleagues could show a relatively good visualization of the capitellum with approximately 66.5% outlined during a standard arthroscopy, so far, no studies have presented the accessibility of the capitellar surface for example in nano-drilling, when more flexible instruments like K-wires are used [24]. This way our study contributed to the current literature as it displays the accessibility during arthroscopy and not only visualization and it presents a preoperative evaluation method as explained in detail in the materials and method section using sagittal MRI or CT scans. If the lesion is within the calculated corridor of 51.3°, the possibility of arthroscopic treatment is only limited using standard equipment. If this option for surgical treatment is used, the patient must also be informed and educated about the possibility of an open approach.

To provide a remedy, angled or reversed instruments could be used to reduce the corridor that is not reachable during elbow arthroscopy, the evaluation should be part of further studies. Same accounts for additional portals that could be applied. However, in this case, the proximity to neurovascular structures could lead to an increased risk for complications. Nonetheless, changing to an open procedure could be necessary and should always be part of the informed consent discussion. To our knowledge, this is the first study of its kind evaluating the accessibility of osteochondrosis dissecans lesions on the elbow during routine arthroscopy.

We acknowledge the limitations of the study. In our observation, we used fresh frozen specimens, but as we used a comparable clinical setup to a real arthroscopy, we consider the differences negligible. Moreover, our sample size was limited. Therefore, further clinical studies should evaluate the accessibility of the anterior capitellar surface.

## Conclusion

Keeping the possibly of a non-reachable capitellar lesion in arthroscopic interventions in mind, surgeons should evaluate their treatment options accordingly and a preoperative evaluation of the lesion’s location should be performed throughout.

## Data Availability

The datasets used and analysed during the current study are available from the corresponding author on reasonable request.
